# The Fuzzy Logic of MicroRNA Regulation: A Key to Control Cell Complexity

**DOI:** 10.2174/138920210791616707

**Published:** 2010-08

**Authors:** Andrea Ripoli, Giuseppe Rainaldi, Milena Rizzo, Alberto Mercatanti, Letizia Pitto

**Affiliations:** 1 Fondazione Toscana “Gabriele Monasterio”, Pisa, Italy; 2 Institute of Clinical Physiology, Pisa, Italy; 3 Istituto Toscano Tumori, Firenze, Italy

**Keywords:** microRNA, Fuzzy Logic, cognitive map, regulatory circuitries.

## Abstract

Genomic and clinical evidence suggest a major role of microRNAs (miRNAs) in the regulatory mechanisms of gene expression, with a clear impact on development and physiology; miRNAs are a class of endogenous 22-25 nt single-stranded RNA molecules, that negatively regulate gene expression post-transcriptionally, by imperfect base pairing with the 3’ UTR of the corresponding mRNA target. Because of this imperfection, each miRNA can bind multiple targets, and multiple miRNAs can bind the same mRNA target; although digital, the miRNAs control mechanism is characterized by an imprecise action, naturally understandable in the theoretical framework of fuzzy logic.

A major practical application of fuzzy logic is represented by the design and the realization of efficient and robust control systems, even when the processes to be controlled show chaotic, deterministic as well unpredictable, behaviours. The vagueness of miRNA action, when considered together with the controlled and chaotic gene expression, is a hint of a cellular fuzzy control system.

As a demonstration of the possibility and the effectiveness of miRNA based fuzzy mechanism, a fuzzy cognitive map -a mathematical formalism combining neural network and fuzzy logic- has been developed to study the apoptosis/proliferation control performed by the miRNA-17-92 cluster/E2F1/cMYC circuitry.

When experimentally demonstrated, the concept of fuzzy control could modify the way we analyse and model gene expression, with a possible impact on the way we imagine and design therapeutic intervention based on miRNA silencing.

## INTRODUCTION

RNA interference is a post-transcriptional mechanism that contributes to a smooth and effective regulation of gene expression. Mediators of RNA interference are miRNAs, a class of endogenous 22-25 nt single-stranded RNA molecules, able to negatively regulate protein coding genes by interfering with mRNA’s original instruction; miRNAs suppress gene expression *via *imperfect base pairing to the 3’ untranslated region (3’-UTR) of target mRNAs, leading to repression of protein synthesis or mRNA degradation [1-5]. 

Up to date, about 700 miRNAs have been identified [6, 7], and a large amount of transcribed genome, as well as the small fraction of protein coding RNA, suggests that this number is going to increase; miRNAs are highly conserved among species and display distinct temporal and spatial pattern expression. It has been computationally evaluated that about one third of human genes are potentially regulated by miRNAs; each of them potentially interacts with several mRNAs, with different level of effectiveness in repression, according to the specific base pairing to the 3’ UTR of the messenger RNA. 

It has been shown that the cell dynamics, arising from the connectivity of genes and proteins, is extremely complex, showing regular as well as chaotic and hyper-chaotic regimes [8, 9], unpredictable whenever deterministic. Nonetheless, such a rich portfolio of behaviours is finely regulated throughout the life of the cell, in response to the change in the environment, in order to guarantee stability and adaptation capability of tissues and organs.

In this amazing scenario, miRNAs seem to play an important role. The behaviour of miRNAs, although digitally coded, appear to be vague; as a consequence of the imperfect base-pairing mechanism, the same molecule, in different contexts, is able to drive the cell towards different phenotypes. The cell environment can modulate the massive action of miRNA molecules, promptly expressed: miRNAs are natural candidates to a central role in cellular adaptability mechanisms.

The same imprecise, fuzzy mechanism is observed in the interaction between transcription factor and gene promoter [10]. In any case, the potentially greater number of targets of a single miRNAs makes potentially higher the vagueness of miRNA behaviour.

## A FUZZY LOGIC APPROACH

miRNAs act vaguely in the complex cell scenario, addressing our thoughts towards fuzzy logic. In the theoretical framework of fuzzy logic [11], vagueness and complexity are strictly tied concepts.

At the basis of the fuzzy logic approach to complexity is Lofti Zadeh's principle of incompatibility, which states: “as the complexity of a system increases, our ability to make precise and yet significant statements about its behaviour diminishes, until a threshold is reached beyond which precision and significance become almost mutually exclusive characteristics” [12]. Reported into a discourse about regulation, Zadeh's statement tells us that the greater the complexity of the processes to be controlled, the greater has to be the imprecision, the vagueness, of an efficient control mechanism. Not surprisingly, the main engineering application of fuzzy logic is the design of control systems, adaptive and robust to noise, such as those operating in airplanes and in home electronic devices.

If Zadeh's statement is true for a washing machine, all the more so for a cell. The vagueness of miRNAs suggest a major role for these molecules in the effective regulation of the noisy, crowded, complex cellular environment, in response to changes in the noisy, crowded and complex extracellular medium.

In the light of fuzzy logic, we can think about gene regulation as a decisional process in a complex system, and use AI methods, rather than differential equations, to develop meaningful models of such processes. A relatively new symbolic method to treat complexity, following the principle of “decreasing precision, increasing intelligence”, is represented by the fuzzy cognitive map.

## THE FUZZY COGNITIVE MAP

A fuzzy cognitive map (FCM) is a soft computing tool, resulting from the combination of fuzzy logic and neural networks [13]; it offers a flexible and powerful framework for representing human knowledge and reasoning. An FCM is an “in silico” model of a system, whose behaviour is described in terms of “concepts” and causal relations among concepts, by means of a fuzzy signed direct graph with feedback. The nodes of the graph are the concepts involved in the description of the behaviour of the modelled system; the signed and weighted arcs among nodes represent the causal relationship that exists among concept. The value of each one of the concepts of the FCM belongs in the range (0, 1), indicating the fuzzy membership of the activation of the concept. The weights of the arcs take values in the set {-1, 0, +1}, where +1 stands for a positive causal connection, -1 for a negative connection, and zero for the absence of a causal connection. In relation with a microarray based study, a link of weight +1 stands for up regulation, while a -1 link means down regulation. Given its matrix-based formulation, the FCM approach is easily scalable to models with a very large number of links, and then particularly suitable to understand the big picture of a systems biology cartoon.

By merging connectionism and vagueness, the FCM has the ability to specify any model of any complexity, to account for linear or non-linear relations and to allow causal propagation.

FCM is a useful tool for modelling, managing and controlling any complex system. It has been used in engineering for modelling supervisory systems, in biology for modelling regulatory and metabolic networks, in social sciences for modelling the behaviour of individuals [14]; in every case, the dynamic of the systems has been studied and fixed equilibrium points, limited cycles, as well as chaotic behaviours have been reproduced.

### An Example: the miRNA-17-92 Cluster/E2F/cMYC Circuitry

**Fig. (1):**
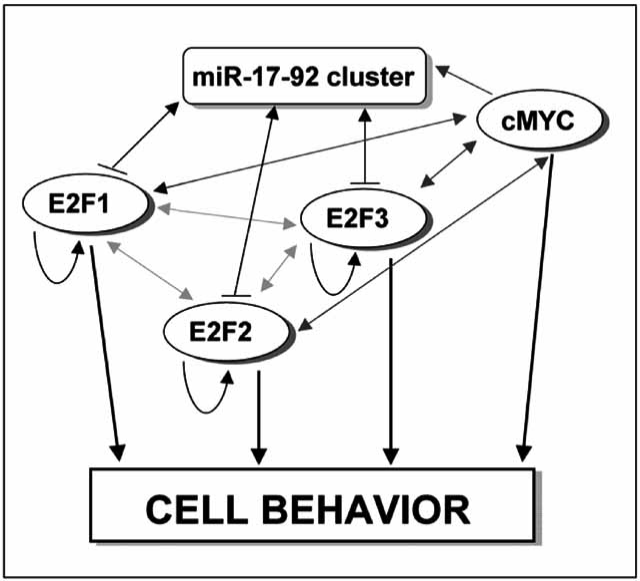
**Schematic representation of Fuzzy Cognitive Map of mRr-17-92/E2Fs/cMYC circuitry.** The miR-17-92 controls cMYC in a self regulating circuit whereby cMYC binds the promoter of the miR-17-92 and increases its transcription In turn miRNAs of the miR-17-92 target E2Fs (E2F1, E2F2 and E2F3) which are able to activate both the cluster and cMYC. In other words, cMYC, while directly increasing the transcription of E2F1, indirectly decreases its translation by inducing miR-17-92 cluster. According to FCM, 6 nodes were identified (cMYC, miR-17-92 cluster, E2Fs and cell behaviour) and the weight of the links were set imposing an unitary positive or negative action on each concept of the map. ( → ) activation; ( ↔ ) reciprocal activation; ( 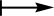 ) arrow indicates activation and vertical bar inhibition.

As a demonstrative case of application of FCM to miRNA regulation, we selected the well studied miRNA-17-92 (miR-17-92)/E2F1/cMYC circuitry (Fig. **1**) [15-17]. This case appeared to be particularly suited for our aims, given the presence in the circuitry of cMYC, an important transcription factor, and the possibility of comparing our results to published ones, obtained with the classical mathematical approach.

It has been reported that miR-17-92 acts as an oncogene or as a tumour suppressor, depending on the transcriptional activity of E2F or cMYC [18]. This conclusion was reached by means of the bifurcation analysis applied to a set of dimensionless differential equations describing protein translation and miRNA transcription, in a simplified circuit. Our model described the whole circuitry, and the weights of the links were set by imposing a unitary positive or negative action on each concept of the map [13]. We introduced a node to express the concept of cell behaviour, and arbitrarily divided the range of its values in zones representing different cellular conditions: quiescence (range: 0-0.25), normal cell cycle (range: 0.25-0.50), proliferation (range: 0.50-0.75) and apoptosis (range: 0.75-1). The value of this node was directly conditioned by the expressions of cMYC, E2F1, E2F2 and E2F3. 

Independently of the initial values chosen for the 6 nodes of the map, the cellular behaviour node indicated a normal cell cycle condition, with mild over-expression of cMYC and miR-17-92 (cell behaviour=0.41, cMYC=0.65, miR-17-92=0.72, E2F1=0.52, E2F2=0.52, E2F3=0.52).

**Fig. (2). Perturbations of FCM and cellular outcomes.**
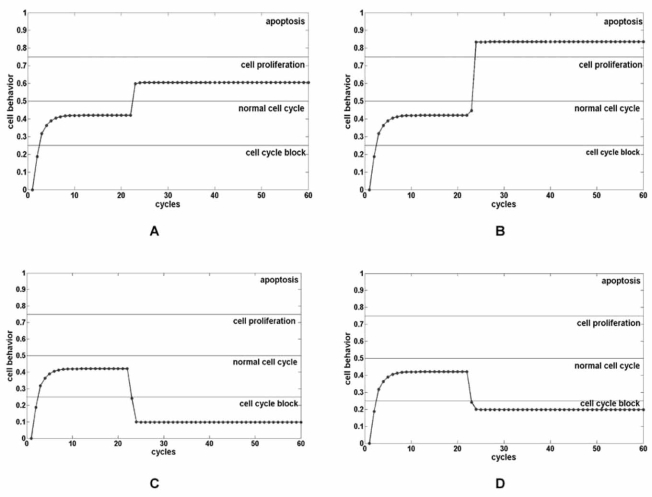
The node cell behaviour can be arbitrarily divided in zones representing cell cycle block (range: 0-0.25), normal cell cycle (range: 0.25-0.50), cell proliferation (range: 0.50-0.75) and apoptosis (range: 0.75-1.0). The value of cell behaviour node is directly conditioned by the expression of cMYC and E2Fs. Independently from the initial values chosen for the 6 FCM nodes, the cell behaviour node indicated a normal cell cycle condition (0.41) and a mild over expression of cMYC (0.65), miR-17-92 (0.72), E2F1 (0.52), E2F2 (0.52) and E2F3 (0.52). By increasing cMYC from 0.65 to 1.0, cells were addressed toward cell proliferation zone (**A**). By decreasing miR-17-92 from 0.72 to 0 cells were addressed toward apoptosis zone (**B**). By increasing miR-17-92 from 0.72 to 1.0 cells were addressed toward cell cycle block zone (**C**). By decreasing cMYC from 0.65 to 0 cells were addressed toward cell cycle block zone (**D**).

Successively, we separately simulated a perturbation in the expression of cMYC and miR-17-92, and we observed their impact on cell behaviour. In every case, the system reached a fixed equilibrium point. Starting from a cell with normal behaviour, a marked increase in cMYC expression (from 0.65 to 1) was not able to drive the cell to apoptosis (Fig. **2A**), condition obtained by abolishing the expression of miR-17-92 (Fig. **2B**). The same results were obtained for the opposite passage in cellular behaviour: the increase of miR-17-92 expression (from 0.72 to 1.0), resulted in a “deeper cell cycle block” (Fig. **2C**), if related to cell cycle block obtained with vanishing cMYC expression (Fig. **2D**).

Globally, even considering the arbitrary subdivision of the cell behaviour in different phenotypes, the simulations showed a great cellular sensitivity to miR-17-92 variation, greater than the one observed for cMYC variations, suggesting its crucial role in the regulation of cellular phenotype, even if there was no direct link in the map between the miRNA node and the cell behaviour node.

## DISCUSSION

In this work we have pointed out the imprecise behaviour of miRNAs and we have suggested that such a vagueness could be a major contribute of miRNAs to gene expression regulation. 

In fact, we showed the effectiveness of a fuzzy logic approach in modelling a miRNA-driven genomic circuitry. The proposed FCM tool resulted more expressive than the simplified mathematical model [18], being able to precisely capture all the range of cellular behaviours, including genomic information and explicitly accounting for the imprecision of regulatory mechanisms.

The mirroring of the imprecision of the molecular mechanism in the imprecision of the modelling approach could doubly inspire the way we imagine and design therapeutic intervention based on miRNA silencing: in the wet-lab, when we introduce new molecules inside cell, and in-silico, when we simulate the complex regulatory processes that are the basis of life and, frequently, of disease.
